# Inhibitory effect of aqueous dandelion extract on HIV-1 replication and reverse transcriptase activity

**DOI:** 10.1186/1472-6882-11-112

**Published:** 2011-11-14

**Authors:** Huamin Han, Wen He, Wei Wang, Bin Gao

**Affiliations:** 1 CAS Key Laboratory of Pathogenic Microbiology and Immunology (CASPMI), Institute of Microbiology, Chinese Academy of Sciences, Beijing, PR China; 2 Graduate University of Chinese Academy of Sciences, Beijing, PR China; 3 School of Life Sciences, University of Science and Technology of China, Hefei 230027, China; 4 China-Japan Joint Laboratory of Molecular Immunology and Microbiology, Institute of Microbiology, Chinese Academy of Sciences, Beijing, PR China; 5 Biochemistry Teaching and Research office of Hebei medical university, Shijiazhuang, PR China

## Abstract

**Background:**

Acquired immunodeficiency syndrome (AIDS), which is caused by the human immunodeficiency virus (HIV), is an immunosuppressive disease that results in life-threatening opportunistic infections. The general problems in current therapy include the constant emergence of drug-resistant HIV strains, adverse side effects and the unavailability of treatments in developing countries. Natural products from herbs with the abilities to inhibit HIV-1 life cycle at different stages, have served as excellent sources of new anti-HIV-1 drugs. In this study, we aimed to investigate the anti-HIV-1 activity of aqueous dandelion extract.

**Methods:**

The pseudotyped HIV-1 virus has been utilized to explore the anti-HIV-1 activity of dandelion, the level of HIV-1 replication was assessed by the percentage of GFP-positive cells. The inhibitory effect of the dandelion extract on reverse transcriptase activity was assessed by the reverse transcriptase assay kit.

**Results:**

Compared to control values obtained from cells infected without treatment, the level of HIV-1 replication and reverse transcriptase activity were decreased in a dose-dependent manner. The data suggest that dandelion extract has a potent inhibitory activity against HIV-1 replication and reverse transcriptase activity. The identification of HIV-1 antiviral compounds from *Taraxacum officinale *should be pursued.

**Conclusions:**

The dandelion extract showed strong activity against HIV-1 RT and inhibited both the HIV-1 vector and the hybrid-MoMuLV/MoMuSV retrovirus replication. These findings provide additional support for the potential therapeutic efficacy of *Taraxacum officinale*. Extracts from this plant may be regarded as another starting point for the development of an antiretroviral therapy with fewer side effects.

## Background

Human immunodeficiency virus type 1 (HIV-1) is the causative agent of acquired immunodeficiency syndrome (AIDS). This disease represents a huge concern for global public health. Currently, there is no effective vaccine for HIV-1 [1]; thus, prevention and antiviral drugs are the only option to decrease morbidity and mortality in HIV-1-infected individuals. Several classes of antiretroviral drugs have been developed targeting viral proteins at different stages of the HIV-1 life cycle or host factors. Highly active antiretroviral therapy (HAART) [2], which typically utilizes a protease inhibitor in combination with a nucleoside and/or nonnucleoside reverse transcriptase inhibitor, is commonly used to treat HIV-1 infected patients. The general problems in current HIV therapy are the constant emergence of drug-resistant HIV strains, adverse side effects and the unavailability of treatments in developing countries. Thus, developing cost-effective, highly-specific and antiresistant drugs are in urgent need.

Natural products, especially those derived from plants, have long been recognized as excellent sources of new anti-HIV-1 drugs. Some of them exhibit inhibitory activity against several HIV-1 processes, including viral entry, reverse transcription, replication, integration, virus maturation, and virion budding. Some of these compounds have been clinically tested, with favorable results [3-8]. Limonoid and nomilin, which are isolated from the seed of *Citrus bergamia*, inhibit both HTLV-1 and HIV-1 reverse transcriptase (RT) activities [9]. Betulinic acid, a triterpenoid isolated from the methyl alcohol extract of the leaves of *Syzigium claviflorum*, and novel betulinic acid derivatives have been used as potent anti-HIV agents [10,11], and new mechanisms for these compounds have been identified [12-14]. These compounds have become a new class of anti-HIV drugs. Phase I and II studies have reported that single oral dose of bevirimat, derived from a betulinic acid-like compound, was well tolerated and demonstrated a dose-dependent reduction in viral load. Derivative IC9564 also competed with gp120/CD4 complexes for binding to chemokine receptors, thus acting as an entry inhibitor that can potently inhibit a broad spectrum of primary HIV-1 isolates by targeting the V3 loop of gp120. Similarly, Trigonostema xyphophylloides from *Euphorbiaceae *and Vatica astrotricha from *Dipterocarpaceae *have been shown to act as HIV-1 entry inhibitors. These compounds likely inhibit HIV-1 replication by blocking the interaction between gp120 and CD4/CCR5 or gp120 and CD4/CXCR4. Petroleum ether from *Rhus chinensis *does not inhibit HIV-1 recombinant RT or HIV-1 entry into host cells, but may target new sites of HIV-1 replication [15]. Shikonin from the dried root of *Lithospermum erythrorhizon *is a pan-chemokine receptor inhibitor [16]. Lipophosphoglycan (LPG) from *Leishmania donovani *has been shown to inhibit the early stage of the HIV-1 life cycle by influencing the membrane fluidity of target cells and diminishing both the virus-cell and cell-to-cell fusion processes initiated by HIV-1 [17].

*Taraxacum officinale*, also known as the common dandelion, has been shown to have heat clearing and detoxifying effects in addition to the ability to eliminate stagnation, remove stasis and induce diuresis for treating strangurtia. This plant is one of the common antiviral agents used in traditional Chinese medicines. Historically, it has been used to treat numerous diseases, ranging from infectious diseases; tumors of the breast, uterus and lung; kidney disease; digestive diseases; and diabetes. Some of the traditional applications of dandelion extract are supported by pharmacological investigation. Modern pharmacological research suggests this plant has broad-spectrum antibacterial [18], anti-fungal [19], antiviral, antidiabetic, choleretic, antirheumatic, anti-inflammatory [20,21], hepatoprotective [22], diuretic [23], and tumor apoptosis-inducing properties [24-26]. However, the antiretroviral properties of this plant have not been examined until recently.

Virus cell-based fluorescence assay using pseudotype particles is an efficient and cost-effective screening system and has been used for primary screening of novel agents against HIV-1 [27-30]. Pseudotyped viruses produced in this system can mimic most stages of the HIV-1 life cycle, including viral protein production, assembly, release, maturation, entry, integration and replication without producing replication-competent viruses. This approach has the potential to identify inhibitors against multiple viral and cellular functions essential for HIV replication [30]. The level of HIV-1 replication has been assessed by the expression of reporter genes represented by the percentage of GFP-positive cells.

This study aimed to investigate the *in vitro *inhibitory effects of aqueous dandelion extract (DWE) on HIV-1 replication by utilizing VSV-G pseudotyped viruses to infect non-CD4 cells. Reverse transcriptase assay kit was used to detect inhibitory effect on RT enzyme activity of dandelion. These results suggest that dandelion extract has a potent inhibitory activity against HIV-1 replication and RT activity. The further investigation should include the identification of the active components in the dandelion extract.

## Methods

### Cell lines and cell culture

All cell lines, the mouse fibroblast cell line (NIH/3T3), 293T human renal epithelial cell, RetroPackPT67 packaging cells and Phoenix Eco packaging cells were purchased from ATCC (Rockville, MD, USA) and grown in Dulbecco's modified eagle medium (DMEM; GIBCO, USA) supplemented with 10% fetal bovine serum (FBS; Gibco, USA), 100 IU/ml penicillin and 100 μg/ml streptomycin. The cells were incubated in a humidified environment with 5% CO_2 _at 37°C. Peripheral mononuclear blood cells (PBMCs) were purified using Ficoll density gradient centrifugation from healthy donors supplied by the Beijing Blood Bank. PBMCs were resuspended and cultured in RPMI-1640 medium supplemented with 10% FBS and 100 U/ml recombinant human IL-2 overnight before they were used in the cytotoxicity assay. Based on an informed consent, this project was approved by the Biomedical Research Ethics Committee of CAS Key Laboratory of Pathogenic Microbiology and Immunology (NO.CASPMI009).

### Retroviral vector and packaging cell construction

pLNCX2 contains elements derived from the Moloney murine leukemia virus (MoMuLV) and the Moloney murine sarcoma virus (MoMuSV) and is designed for retroviral gene delivery. The pLNCX2 retroviral vector (donated by professor Huang) was chosen to insert enhanced green fluorescent protein (EGFP). The EGFP gene was amplified by PCR using the following primers: EGFP-F, CCGCTCGAGGCCGCCAC CATGGTGAGCAAGGGCGAGGAGCT; EGFP-R, ACGCGTCGACCTACTTGTA CAGCTCGTCCATGCCGAGAGAGT, and the fragment was inserted into the pLNCX2 retroviral vector at the XhoI/Sal I restriction enzyme site.

The Phoenix Eco packaging cells were transfected with pLNCX2-EGFP using calcium phosphate-based transfection. The retroviral supernatant was collected 48 h post-transfection, filtered through a 0.45 μm filter and stored at -80°C for subsequent use. RetroPack PT67 cells were subsequently infected with the retrovirus-containing supernatant in the presence of 8 μg/ml polybrene (Sigma Co., St Louis, MO) using centifugation at 1200 xg at 32°C for 90 min. Six hours after infection, the supernatant was removed and the cells were incubated in the complete medium. The expression of the pLNCX2-EGFP retroviral vector was verified by selection with 100 μg/ml G418 (GIBCO, USA).

The pLNCX2-EGFP-transfected RetroPack PT67 packaging cells encoding the EGFP genes were used as a stable EGFP virus-producing cell line and cultured in DMEM supplemented with 10% FBS. Retaining one plate for the continuation of the culture, the remaining cells were plated at 60-80% confluence in the desired number of culture vessels. Viral supernatants could then be harvested in 24 h intervals until the cells were no longer viable. Once the virus was harvested, all cells were discarded.

### Production of pseudotyped virus and transduction

To produce single-cycle infectious virons, VSV-G pseudotyped HIV-1 vector stocks were prepared, concentrated and titered as described previously [31]. The pLL3.7 plasmid (6 μg; Invitrogen, USA), belonging to the third generation of lentivirus vector system based on HIV-1, was co-transfected with pLP1 (Invitrogen, USA), pLP2 (Invitrogen, USA) and pLP/VSVG (Invitrogen, USA) into 293T cells using calcium phosphate-based transfection. After transfection (48 to 72 h), the virus-containing supernatants were harvested, passed through a 0.45 μm filter and frozen in aliquots at -80°C until use. NIH/3T3 cells were placed in each well of a six-well plate with 2 ml DMEM containing 10% FBS. The next day, the media was replaced with 2 ml of virus supernatants containing 8 μg/ml of polybrene at a multiplicity of infection (MOI) of 1 in the presence or absence of DWE. Zidovudine (AZT) and *Herba Artemisiae Scopariae *were used as positive control and negative control respectively. The cells were infected by spinoculation in a centrifuge at 1, 200 xg for 90 min at 32°C and incubated at 37°C overnight, followed by a change to fresh medium.

### Plant material

Samples of the entire plant of *Taraxacum officinale *(dried) and *Herba Artemisiae Scopariae *(dried) were purchased at Fenlinlvzhou medicinal store in Beijing, China in December 2010 and authenticated by ph.D Wei Li, Institute of Microbiology, Chinese Academy of Sciences. The voucher specimen of these plant materials were deposited in the CAS Key Laboratory of Pathogenic Microbiology and Immunology (CASPMI), Institute of Microbiology, Chinese Academy of Sciences.

### Quantification of the inhibitory effect of DWE to HIV-1 RT

The effect of DWE on HIV-1 RT activity was evaluated with the reverse transcriptase assay, coloremetric kit (Version 13.0, Roche, USA). The assay was performed according to the manufacture's instructions by transferring 20 μl of recombinant HIV-1 RT and 20 μl reaction buffer to microfuge tubes containing different concentrations of DWE (2 mg/ml, 1 mg/ml, 0.75 mg/ml, 0.5 mg/ml, 0.25 mg/ml, 0.125 mg/ml) and diluted with lysis buffer. 2.5 μM AZT was used as a positive control, lysis buffer without HIV-1 RT was used as one negative control (control 1), lysis buffer with HIV-1 RT but no DWE was used as another negative control (control 2). After 1 h incubation at 37°C, the samples were transfered into microplate modules for 1 h incubation again at 37°C. The plate was rinsed 5 times with 250 μl of washing buffer and buffer was completely removed before adding 200 μl of anti-DIG-POD working dilution. A further 1 h incubation at 37°C followed by washing 5 times with washing buffer. 200 μl ABTS substrate solution was added per well and incubated at room temperature until color development is sufficient for photometric detection (10-30 min). The absorbance was read on a microplate reader at 405 nm and a reference wavelength of 490 nm. The assay was carried out in triplicate and repeated three times. Results were analyzed using the formula:

$$\%\text{ relative inhibition }=\;\left(\text{R}{\text{T}}_{\text{control 2}}-\text{R}{\text{T}}_{\text{control 1}}\right)-\left(\text{R}{\text{T}}_{\text{sample}}-\text{R}{\text{T}}_{\text{control 1}}\right)\times \text{1}00∕\text{ R}{\text{T}}_{\text{control 2}}-\text{R}{\text{T}}_{\text{control 1}}$$

### Preparation of extracts

10 g of the dried whole plant of *Taraxacum officinale *and *Herba Artemisiae Scopariae *were respectively soaked in water for 0.5 h at room temperature. Hot water extractions were performed at 100°C in three batches: 200 ml for 3 h, 150 ml for 2 h, 100 ml for 1 h. The mixtures were then pooled together and filtered to remove the particulate matter, concentrated to 100 ml, filtered through a 0.22 μm syringe filter and lyophilized, yielding light yellow power.

### Cell survival assay

The cytotoxicity of DWE to NIH/3T3 cells was assessed using a CCK-8 assay (Cell Counting Kit, Dojindo Laboratories, Kumamoto, Japan). NIH3T3 cells were seeded in 96-well plates at an initial density of 3 × 10^3 ^cells/well in 100 μl of culture medium. After 24 h of incubation, the cells were treated in the presence or absence of the different compounds at different concentrations ranging from 8 mg/ml to 0.25 mg/ml and 100 μM to 0.0001 μM of AZT. The medium was removed, and the cells were washed twice with fresh media. Next, 100 μl of fresh serum-free DMEM containing 1/10(v/v) Cell Counting Kit-8 reagent was added to each well and incubated for an additional 4 h. After incubation, the viability of the NIH/3T3 cells was assayed with CCK-8 using a 96-well plate reader (DG5032, Huadong, Nanjing, China) at 450 nm. Untreated cells were served as the negative control, and wells containing the Cell Counting Kit-8 reagent and no cells were used as the blank control. Cytotoxicity was assessed by the cell survival rate. The absorbance reading from each well was used to calculate the cell survival rate. Survival rate (%) = optical density (OD) of the treated cells -OD of blank control/OD of negative control-OD of blank control × 100.

The cytotoxicity of DWE in human PBMCs was determined using the trypan blue exclusion method. Briefly, PBMCs were cultured at 1.3 × 10^6 ^cells/ml in a 24-well plate supplemented with 100 IU/ml IL-2 and exposed to increasing concentrations of DWE for 24 h. PBMCs without DWE treatment were used as a control. The cells were stained with 0.2% trypan blue dye, and the total cells and viable cells were counted under a light microscope.

### Flow cytometry, fluorescence microscopy analysis and image acquisition

NIH3T3 cells were harvested using a trypsin/EDTA solution, washed with ice-cold PBS three times and resuspended in PBS to a final concentration of 10^6 ^cells/ml. Virus infection was measured in the absence or presence of increasing concentrations of DWE. The percentage of EGFP-positive cells was used to calculate the infection efficiency by flow cytometry using Guava EasyCyte (Guava Technologies, USA). All FACS data were analyzed using FlowJo software (Tree Star, Ashland OR). Images were acquired on a ZEISS Axiovert 200 M microscope (Zeiss, Vienna, Austria) with a 40 x/0.55NA objective. The microscope and image acquisition were controlled by AxioVision software. For GFP observation, we used blue light excitation. Image processing, such as the clipping of images, was first performed in PowerPoint and then converted to TIFFs.

### High performance liquid chromatography(HPLC)

200 mg freeze-dried aqueous extract powder was dissolved in 2 ml milli-Q water, and filtered through a 0.22 μm syringe filter before analysis. HPLC separation was conducted by a Shimadzu CTO-15C system equipped with a binary high pressure gradient pump, UV/Vis detector SPD-15C. Peak areas were calculated with a LC solution 15C software. Gradient elution was performed using two solvents consisting of A (0.1% (v/v) TFA in H_2_O) and B ((0.1%(v/v) TFA in acetonitrile). Step gradient is from 5% to 70% of B over 40 min. Total chromatography duration was 40 min. The flow-rate of mobile phase was 1 ml/min. Injection volume was 25 μl. The detection wavelengths were set at 254 and 323 nm, and column temperature was room temperature. The caffeic acid and chlorogenic acid purchased from Sigma (USA) were used as the reference standards for quantitative assay. The concentrations of components present in DWE were identified by comparing chromatographic peaks with the retention time of individual standards.

### Statistical analysis

Data are presented as mean ± SD. The data were statistically evaluated using a one-way ANOVA to compare differences between the groups. A p-value of < 0.05 was considered to be significant. The IC50 values were calculated using GraphPad Prism programme.

## Results

### Production of the VSV-G pseudotyped HIV-1 virus and 10A1 pseudotyped pLNCX2-EGFP vector

**Figure 1:**
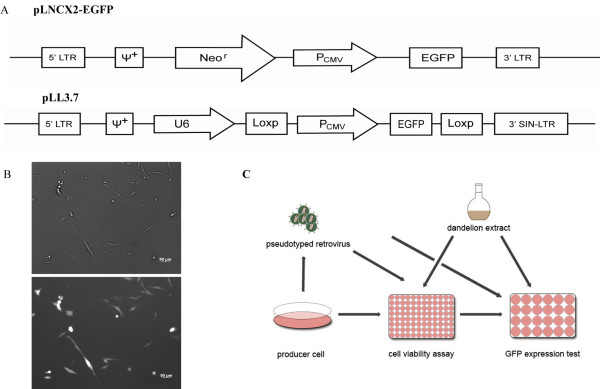
**Construction of retroviral and HIV-1 replication reporter vector system**. (A) The pLNCX2 -EGFP vector, constructed by inserting the EGFP gene into the multiple cloning site of the pLNCX2 plasmid, a retroviral vector contains elements derived from Moloney murine leukemia virus (MoMuLV) and Moloney murine sarcoma virus (MoMuSV). The HIV-1 based lentiviral vector pLL3.7 is commercially available, and EGFP is driven by the CMV promoter. (B) The development of stable RetroPackPT67 packaging cells encoding the EGFP genes, which was used as the EGFP virus-producing cell line. (C) Outline of the anti-HIV-1 activity assay for DWE.

We chose EGFP as a reporter gene because its expression can be easily monitored. As illustrated in Figure 1A, expression of EGFP served as an indicator of viral replication. pLNCX2-EGFP was generated by inserting the EGFP gene downstream from the CMV promoter. Virus-producing RetroPack PT67 cells were established through a ping-pong method, which uses the virus from the Eco packaging cell line to infect RetroPack PT67 cells. Finally, a stable pLNCX2-EGFP transfected RetroPack PT67 cell line was selected using G418. As shown in Figure 1B, high level of EGFP was observed in all RetroPack PT67 cells. Our experimental design was showned in Figure 1C.

### *In vitro *effects of *Taraxacum officinale *extracts on cell viability

**Figure 2:**
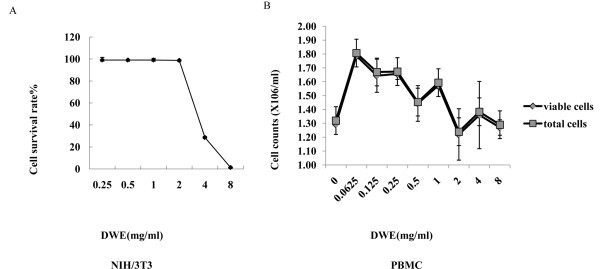
**Cytotoxicity assay for different cells cultured with different concentrations of DWE**. (A) NIH/3T3 cell survival curves from the CCK-8 assay. The negative control was untreated cells, and the blank control contained the Cell Counting Kit-8 reagent and no cells. The curve represents the average of three independent experiments with SD bars. (B) PBMCs were incubated at 37°C in 5% CO_2 _overnight in media supplemented with IL-2 at a concentration of 100 IU/ml. The cells were then exposed to the DWE for 24 h, and aliquots of cells were stained with trypan blue dye to determine the total numbers of cells as well as viable cells. PBMC cells, cultured in the absence of DWE were used as control. These data represent three independent experiments.

The dried whole plant of *T. officinale *was extracted with hot water, yielding DWE (17% w/w). It was important to assess whether the compounds were toxic to exclude a non-specific antiretroviral effect. We determined the effects of the extracts on cell survival in the absence of HIV-1 infection to establish a non-toxic working concentration of the DWE. The cytotoxicity of DWE in NIH/3T3 cells was evaluated using a CCK-8 assay. The experiments were repeated three times for six different concentrations (0.25 mg/ml, 0.5 mg/ml, 1 mg/ml, 2 mg/ml, 4 mg/ml, 8 mg/ml). For concentrations up to 2 mg/ml, no significant cytotoxic effects were found (Figure 2A). The concentration of 2 mg/ml was chosen to carry out the subsequent studies based on cell viability and morphological observations.

### *Taraxacum officinale *extract has no toxicity in human PBMCs

The cytotoxicity of DWE on human PBMCs was tested by evaluating cellular viability and was confirmed using the trypan blue test following exposure to increasing concentrations of DWE (0.0625 mg/ml to 8 mg/ml). As shown in Figure 2B, PBMC viability was not decreased at concentrations up to 8 mg/ml. Compared to untreated PBMCs, both the number of total cells and the number of viable cells were significantly increased after treatment with DWE at a concentration of 0.0625 mg/ml (p < 0.05). These data indicate that DWE exhibits no toxicity on human PBMCs and served as a nutrient or stimulator at a low concentration.

### Inhibitory activity of *Taraxacum officinale *extract on HIV-1 replication

**Figure 3:**
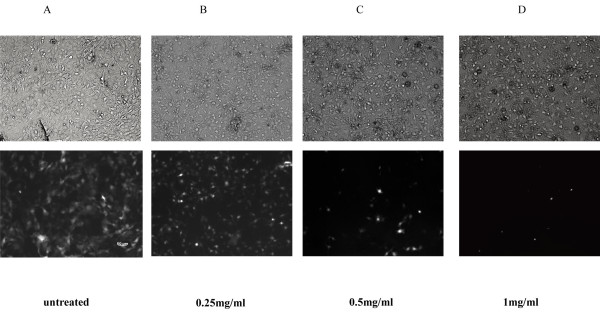
**Fluorescence micrograph of GFP proteins in infected cells treated with DWE**. NIH/3T3 cells expressing EGFP following infection with VSV-G pseudotyped HIV-1 virus. (A) NIH/3T3 cells infected with the pLL3.7 lentivirus without DWE (untreated). Figures show NIH/3T3 cells infected with the pLL3.7 lentivirus in the presence of DWE at a concentration of (B) 0.25 mg/ml (C) 0.5 mg/ml (D) 1 mg/ml. In the upper panels, cells are visualized under normal light, while in the lower panels, the same cells are visualized by fluorescent microscope.

**Figure 4:**
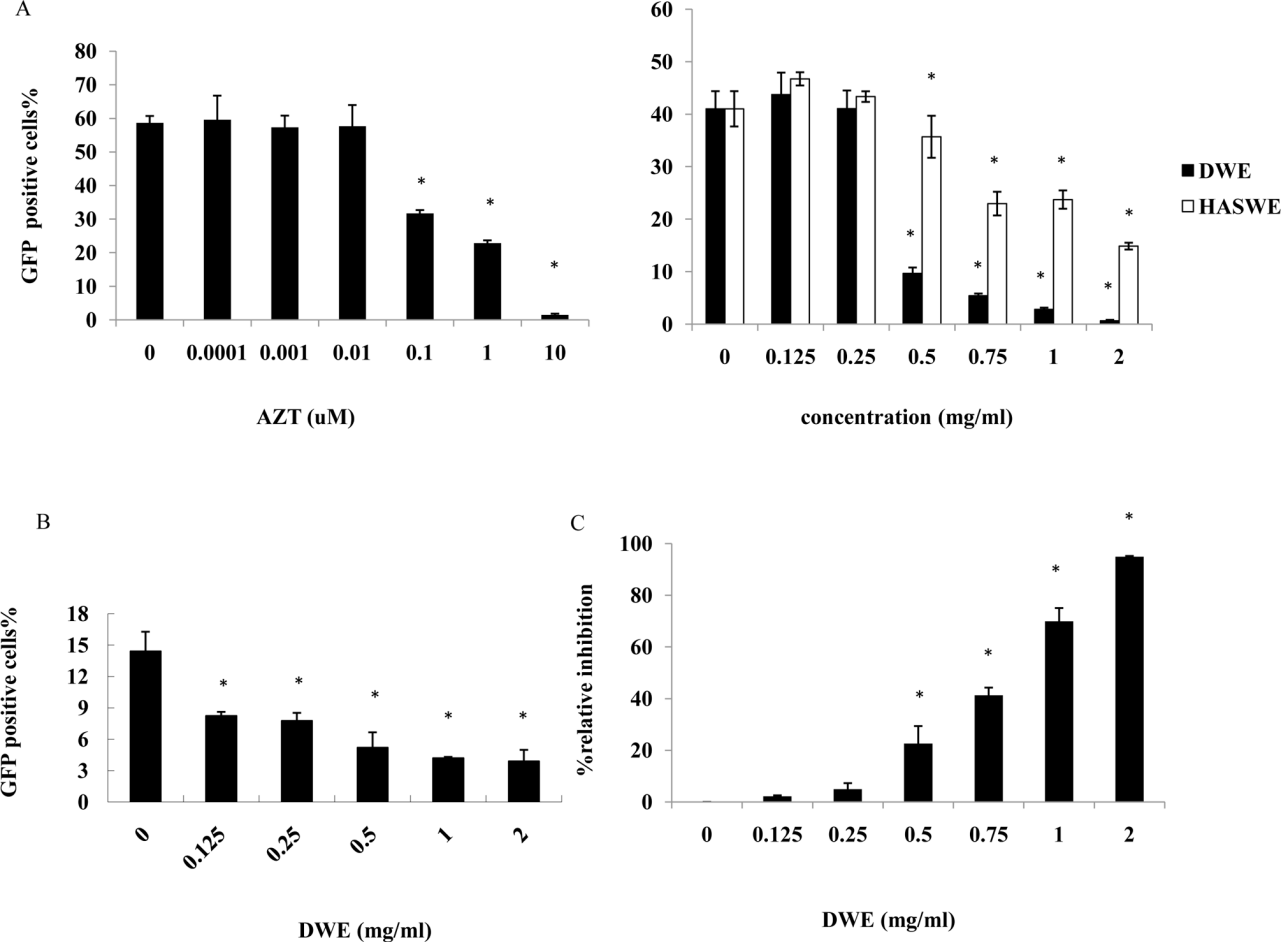
**Inhibitory effects of DWE on HIV-1 and another retrovirus**. Figure A and B shows the effects of DWE on HIV-1 and another retrovirus pLNCX2 -EGFP replication. Gene expression of NIH/3T3 cells were infected with two pseudotyped retroviruses for 12 h and treated with different concentrations of DWE. Different concentrations of AZT (from 0 μM to 10 μM) and aqueous *Herba Artemisiae Scopariae *extract (HASWE) were used as positive control and negative control respectively. (A) NIH/3T3 cells infected with VSV-G pseudotyped HIV-1; (B) NIH/3T3 cells infected with MoMuLV/MoMuSV hybrid retrovirus; (C) Percentage of inhibition of HIV-1 RT by DWE. 2.5 μM AZT was used as the positive control with 98% inhibitory rate while lysis buffer with no HIV-1 RT was used as one negative control, lysis buffer with HIV-1 RT but no DWE was used as another negative control. The data represent the mean ± SD of triplicate experiments

When compared to control values obtained from infected cells that were not treated with the plant extracts, DWE showed inhibitory activity that increased in a concentration-dependent manner. This was seen as a decrease of GFP-positive counts in a dose-dependent manner (see graphs in Figure 3). AZT, a reverse transcriptase inhibitor, was used as a positive control. As shown in Figure 4A, the 50% inhibitory concentration (IC_50_) of AZT for HIV-1 was 0.29 μM, which is in accordance with previously published data. We used the previously determined non-toxic concentration of 2 mg/ml as the starting testing point for DWE. The maximum inhibitory effect (98%) was obtained with 2 mg/ml, and the IC_50 _of DWE was 0.64 mg/ml, As a negative control, the extract from *Herba Artemisiae Scopariae *showed much weaker inhibitory activity with an IC50 of 1.255 mg/ml when compared to the activities of *Taraxacum officinale *observed. Next, we determined whether DWE was similarly effective in the inhibition of another retrovirus unrelated to HIV-1, the 10A1-pseudotyped hybrid-MoMuLV/MoMuSV retrovirus. We found DWE also to be effective for this virus. The maximum inhibitory effect (73%) was obtained with 2 mg/ml, and the IC_50 _of DWE was 0.23 mg/ml (Figure 4B). The similar ability of DWE to inhibit the replication of two unrelated retroviruses suggests that dandelion extract may target a factor that is broadly required for retroviral replication.

### Inhibitory effects of *Taraxacum officinale *extract on HIV-1 RT activity

Anti-HIV-1 activity of the aqueous extract of dandelion was further investigated using RT assay, which is a colorimetric assay where the enzyme activity is determined after treatments in the presence or absence (untreated control) of different concentrations of DWE. Inhibition of DWE to the HIV-1 RT enzyme was evaluated based on their percent inhibition compared to a sample that does not contain DWE. As showned in Figure 4C, DWE showed potent inhibitory effect at 2 mg/ml (94.89%) with an IC50 of 0.87 mg/ml compared to RT inhibitor, AZT at 2.5 μM (98%), and the inhibitory activities were dose dependent.

## Discussion

**Figure 5:**
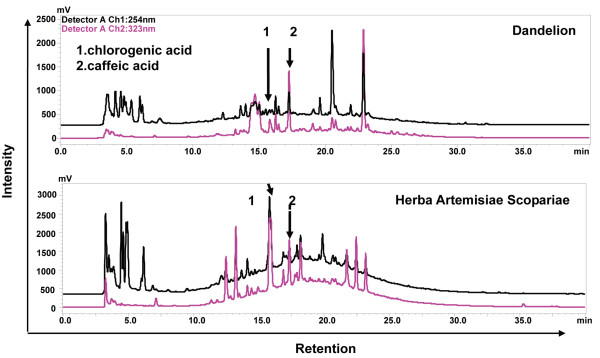
**HPLC chromatograms**. HPLC chromatogram of *Taraxacum officinale *and *Herba Artemisiae Scopariae *which are (1) caffeic acid, (2), chlorogenic acid.

The present study demonstrates for the first time that DWE displays inhibitory effect on HIV-1 replication and RT activitiy. It has been reported that extracts containing marked concentrations of chlorogenic acid inhibited HIV reverse transcriptase [32,33]. Dandelion are rich in phenolic compounds, in particular chlorogenic acid, caffeic acid, various flavonoid glycosides such as luteolin 7-O-glucoside, luteolin and others, however, as shown in Figure 5, HPLC analysis of two Compositae plant species showed that *Herba Artemisiae Scopariae *extract, which contain higher contents of chlorogenic acid and caffeic acid than those in DWE (212-fold and 7-fold, respectively), showed milder inhibitory activity on HIV-1 replication. Since DWE are mixture of components, there is a possibility that the anti-HIV-1 compound may be novel. The isolation of active components against HIV-1 RT from *Taraxacum officinale *is now in progress. The activity exhibited by DWE gives some evidence to validate their effect against HIV-1.

Cell-based assays are one of the high-throughput screening used to identify new inhibitors that target on different steps in the HIV-1 life cycle. Single-cycle infectious pseudotyping of HIV-1 using the envelope glycoprotein of the vesicular stomatitis virus (VSV-G) can mimic some behaviors of the wild-type viruses [34]. High-titer stocks of the single-cycle infectious virus can be generated from producer cells. In addition, using the single-cycle infectious viruses in HIV-1 reporter virus assays significantly reduces the safety risk encountered with replication-competent HIV-1. Taking these into account, experts emphasize the importance of developing cell-based screening systems for the rapid identification of novel anti-HIV agents [35-38]. Moreover, to explore the different mechanism of anti-HIV-1 activity, choosing an appropriate cell line is a critical step. Human CD4+ Jurkat cells are commonly used in screening anti-HIV-1 drugs [6,39,40], but other reports have indicated dandelion root extract shows a selective induction of apoptosis through the activation of caspase-8 in human leukemia cells (Jurkat) [26]. In our study, we used VSV-G pseudotyped HIV-1, which has a different entry pathway from wild-type HIV-1 [34], and a non-CD4 cell line. The assay used here targeted HIV-1 replication, a post-entry event. A deletion in the U3 region results in the loss of promoter activity following reverse transcription and integration. The only promoter that dictates the transcription of a reporter gene is the internal promoter CMV. Thus, the expression of GFP protein is a reliable indicator of HIV-1 replication [41]. It is also important to distinguish specific antiretroviral activity from non-specific inhibitor effects or DWE-mediated cytotoxicity. We combined cytotoxicity data with antiretroviral testing to solve this issue. DWE has been studied in mice, and it was reported that the administration of DWE at 1 g/kg/day for 4 weeks did not produce adverse effects [22]. We also observed that DWE had no cytotoxic effect on primary PBMCs.

## Conclusions

In conclusion, the dandelion extract showed strong activity against HIV-1 RT and inhibited both the HIV-1 vector and the hybrid-MoMuLV/MoMuSV retrovirus replication. These findings provide additional pharmacological information on the potential therapeutic efficacy of *Taraxacum officinale*. This could represent another starting point for the development of an anti-retroviral therapy with fewer side effects.

This preliminary finding suggested to isolate bioactive compound from biologically active extracts for further study. Components activity tests of the plants extracts would be performed for anti-HIV-1 activities. It is desirable to determine the effects of DWE on wild-type HIV-1 as well as to try to isolate active constituents.

## Competing interests

The authors declare that they have no competing interests.

## Authors' contributions

HMH performed experiments and prepared the manuscript. WH and WW performed experiments. BG designed the experiments, supervised the project. All authors read and approved the final manuscript.

## Pre-publication history

The pre-publication history for this paper can be accessed here:

http://www.biomedcentral.com/1472-6882/11/112/prepub
